# High-quality draft genome sequence of *Ensifer meliloti* Mlalz-1, a microsymbiont of *Medicago laciniata* (L.) miller collected in Lanzarote, Canary Islands, Spain

**DOI:** 10.1186/s40793-017-0270-2

**Published:** 2017-09-25

**Authors:** Wan Adnawani Meor Osman, Peter van Berkum, Milagros León-Barrios, Encarna Velázquez, Patrick Elia, Rui Tian, Julie Ardley, Margaret Gollagher, Rekha Seshadri, T. B. K. Reddy, Natalia Ivanova, Tanja Woyke, Amrita Pati, Victor Markowitz, Mohamed N. Baeshen, Naseebh Nabeeh Baeshen, Nikos Kyrpides, Wayne Reeve

**Affiliations:** 1 School of Veterinary and Life Sciences, Murdoch University, Murdoch, WA Australia; 2 U.S. Department of Agriculture, Soybean Genomics and Improvement Laboratory, Beltsville Agricultural Research Center, 10300 Baltimore Avenue, Bldg. 006, Beltsville, MD 20705 USA; 3 Departamento de Bioquímica, Microbiología, Biología Celular y Genética, Universidad de La Laguna, Tenerife, Spain; 4 Departamento de Microbiología y Genetica and Instituto Hispanoluso de Investigaciones Agrarias (CIALE), Universidad de Salamanca, Salamanca, Spain; 5 Curtin University Sustainability Policy Institute, Curtin University, Bentley, WA Australia; 6 DOE Joint Genome Institute, Walnut Creek, CA USA; 7 Biological Data Management and Technology Center, Lawrence Berkeley National Laboratory, Berkeley, CA USA; 8 Department of Biology, Faculty of Science, University of Jeddah, Jeddah, Saudi Arabia

**Keywords:** Root-nodule bacteria, *Ensifer*, Geba-Rnb, *Medicago*, *lpiA-acvB* operon

## Abstract

10.1601/nm.1335 Mlalz-1 (INSDC = ATZD00000000) is an aerobic, motile, Gram-negative, non-spore-forming rod that was isolated from an effective nitrogen-fixing nodule of *Medicago laciniata* (L.) Miller from a soil sample collected near the town of Guatiza on the island of Lanzarote, the Canary Islands, Spain. This strain nodulates and forms an effective symbiosis with the highly specific host *M. laciniata*. This rhizobial genome was sequenced as part of the DOE Joint Genome Institute 2010 Genomic Encyclopedia for Bacteria and Archaea-Root Nodule Bacteria (GEBA-RNB) sequencing project. Here the features of 10.1601/nm.1335 Mlalz-1 are described, together with high-quality permanent draft genome sequence information and annotation. The 6,664,116 bp high-quality draft genome is arranged in 99 scaffolds of 100 contigs, containing 6314 protein-coding genes and 74 RNA-only encoding genes. Strain Mlalz-1 is closely related to 10.1601/nm.1335 10.1601/strainfinder?urlappend=%3Fid%3DIAM+12611 ^T^, 10.1601/nm.1334 A 321^T^ and 10.1601/nm.17831 10.1601/strainfinder?urlappend=%3Fid%3DORS+1407 ^T^, based on 16S rRNA gene sequences. gANI values of ≥98.1% support the classification of strain Mlalz-1 as 10.1601/nm.1335. Nodulation of *M. laciniata* requires a specific *nodC* allele, and the *nodC* gene of strain Mlalz-1 shares ≥98% sequence identity with *nodC* of *M. laciniata*-nodulating 10.1601/nm.1328 strains, but ≤93% with *nodC* of 10.1601/nm.1328 strains that nodulate other *Medicago* species. Strain Mlalz-1 is unique among sequenced 10.1601/nm.1335 strains in possessing genes encoding components of a T2SS and in having two versions of the adaptive acid tolerance response *lpiA-acvB* operon. In 10.1601/nm.1334 strain 10.1601/strainfinder?urlappend=%3Fid%3DWSM+419, *lpiA* is essential for enhancing survival in lethal acid conditions. The second copy of the *lpiA-acvB* operon of strain Mlalz-1 has highest sequence identity (> 96%) with that of 10.1601/nm.1334 strains, which suggests genetic recombination between strain Mlalz-1 and 10.1601/nm.1334 and the horizontal gene transfer of *lpiA-acvB*.

**Electronic supplementary material:**

The online version of this article (10.1186/s40793-017-0270-2) contains supplementary material, which is available to authorized users.

## Introduction

Symbiotic nitrogen fixation by pasture legumes and their associated root nodule bacteria provides a critical contribution to sustainable animal and plant production, and the maintenance of soil fertility in agricultural systems [1–3]. As such, it is of direct relevance to maintaining environmentally sustainable high agricultural yields, which significantly contributes to the Sustainable Development Goals adopted in September 2015 as part of the UN’s development agenda ‘Transforming our world: the 2030 Agenda for Sustainable Development’ [4]. Medics (*Medicago* spp.) are some of the most important and extensively grown pasture legumes and their specific symbiosis with strains of rhizobia belonging to either 10.1601/nm.1328 (synonym 10.1601/nm.1339) *meliloti* or the closely related species 10.1601/nm.1334 [5, 6] has been the subject of extensive research efforts [7].

*Medicago laciniata* (L.) Miller (cut leaf medic), an annual native of southern and eastern Mediterranean and Saharo-Sindian countries, is of importance because of its ability to grow in comparatively arid habitats and marginal cropping areas [8–11]. It is highly specific in its rhizobial requirements, forming a symbiosis only with a restricted subset of 10.1601/nm.1335 and not with strains that nodulate *Medicago sativa* L. (alfalfa) or *Medicago truncatula* Gaertn. [12, 13]. This symbiotic specificity has been linked to the rhizobial *nod* genes, in particular a specific *nodC* allele [14]. For example, van Berkum and colleagues found that most rhizobial strains isolated from Tunisian *M. truncatula* and *M. laciniata* shared chromosomal identity, but differed in their *nodC* alleles [15]. Based on these and other differing symbiotic traits, Villegas et al. [13] proposed two biovars within 10.1601/nm.1335: bv. medicaginis for 10.1601/nm.1328 strains that are symbiotically efficient on *M. laciniata* and bv. meliloti for the classical 10.1601/nm.1335 group that efficiently nodulates *M. sativa*. However, in subsequent studies the diversity observed within bv. medicaginis strains indicate that this group is certainly heterogeneous [16].

*M. laciniata* is native to the Canary Islands and is present on all of the islands of this archipelago, growing in environments that range from arid to subhumid. 10.1601/nm.1335 strain Mlalz-1 was isolated from a N_2_-fixing nodule of *M. laciniata* grown in alkaline soil (pH 9.0) collected in Guatiza, in the arid Northeast of Lanzarote Island, in 2007. This strain was one of the rhizobial genomes sequenced as part of the DOE Joint Genome Institute 2010 GEBA-RNB project proposal [17, 18]. Here an analysis of the complete genome sequence of 10.1601/nm.1335 Mlalz-1 is provided.

## Organism information

### Classification and features

**Fig. 1:**
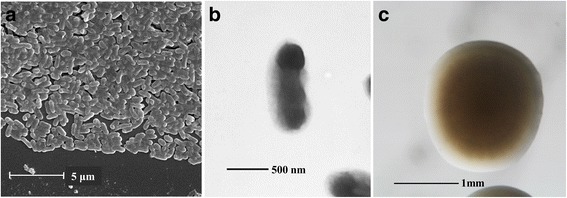
Images of 10.1601/nm.1335 Mlalz-1 using scanning (*Left* (**a**)) and transmission (*Center* (**b**)) electron microscopy as well as light microscopy to visualize colony morphology on solid media (*Right* (**c**))

10.1601/nm.1335 Mlalz-1 is a motile, non-sporulating, non-encapsulated, Gram-negative strain in the class 10.1601/nm.809. The rod shaped form has dimensions of approximately 0.5 μm in width and 1.0–2.0 μm in length (Fig. 1 *Left* and *Center*). It is fast growing, forming colonies after 3–5 days when grown on ½LA, TY, or a modified yeast-mannitol agar [19] at 28 °C. Colonies on ½LA are opaque, slightly domed and moderately mucoid with smooth margins (Fig. 1 *Right*). Minimum Information about the Genome Sequence (MIGS) for strain Mlalz-1 is provided in Table 1 and Additional file 1: Table S1.

**Table 1:** Classification and general features of 10.1601/nm.1335 Mlalz-1 in accordance with the MIGS recommendations [65] published by the Genomic Standards Consortium [66]

MIGS ID	Property	Term	Evidence code^a^
	Current classification	Domain Bacteria	TAS [67]
		Phylum 10.1601/nm.808	TAS [68]
		Class 10.1601/nm.809	TAS [69, 70]
		Order 10.1601/nm.1277	TAS [70, 71]
		Family 10.1601/nm.1278	TAS [72, 73]
		Genus 10.1601/nm.1328	TAS [74]
		Species 10.1601/nm.1335	[21]
		Strain: Mlalz-1 (= 10.1601/strainfinder?urlappend=%3Fid%3DUSDA+1984)	IDA
	Gram stain	Negative	IDA
	Cell shape	Rod	IDA
	Motility	Motile	IDA
	Sporulation	Non-sporulating	NAS
	Temperature range	10–40 °C	IDA
	Optimum temperature	25–30 °C	IDA
	pH range; Optimum	5–9.5; 6.5–8	IDA
	Carbon source	Varied	IDA
MIGS-6	Habitat	Soil; root nodule on host *Medicago laciniata* (L.) Miller	IDA
MIGS-6.3	Salinity	Tolerates 0 to 1% (*w*/*v*) % NaCl	TAS
MIGS-22	Oxygen requirement	Aerobic	IDA
MIGS-15	Biotic relationship	Free living, symbiotic	IDA
MIGS-14	Pathogenicity	Biosafety level 1	TAS [75]
MIGS-4	Geographic location	Guatiza, Lanzarote, Canary Islands, Spain	IDA
MIGS-5	Sample collection date	2007	IDA
MIGS-4.1	Latitude	29.074324	IDA
MIGS-4.2	Longitude	−13.479696	IDA
MIGS-4.3	Depth	5–10 cm	IDA
MIGS-4.4	Altitude	102 m	IDA

^a^Evidence codes – *IDA* Inferred from Direct Assay, *TAS* Traceable Author Statement (i.e., a direct report exists in the literature), *NAS* Non-traceable Author Statement (i.e., not directly observed for the living, isolated sample, but based on a generally accepted property for the species, or anecdotal evidence). Evidence codes are from the Gene Ontology project [76, 77]

#### Symbiotaxonomy

*M. laciniata* is a highly specific host and its microsymbionts also appear to be highly specific since studies of *Medicago* isolates have shown that *M. laciniata* strains fail to nodulate a range of *Medicago* species [5, 12]. Bailly et al. [20] reported that isolates of *M. laciniata* nodulated and fixed nitrogen with *M. truncatula*, but also provided evidence that these were the progeny of horizontal transfer of the nodulation genes. Strain Mlalz-1 nodulates and is effective for nitrogen fixation with *M. laciniata*. We report here that strain Mlalz-1 is unable to nodulate *Medicago polymorpha* L., the definitive host for 10.1601/nm.1334 strains [6].

#### Extended feature descriptions

**Fig. 2:**
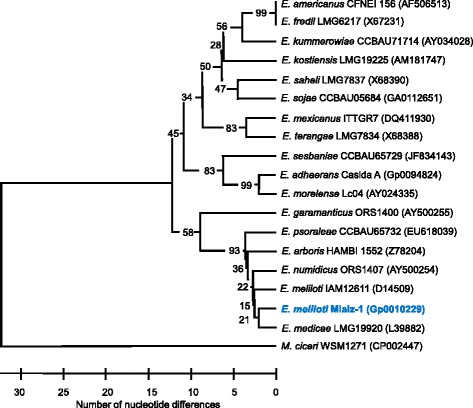
Comparison of the 16S rRNA gene sequences of 10.1601/nm.1335 Mlalz-1 (shown in bold) and other 10.1601/nm.1328 spp. type strains, based on aligned 16S rRNA gene sequences of 1389 bp. Of the 1389 sites, 1279 were constant and 48 were informative. There were eight gaps overall when 10.1601/nm.1418 bv biserrulae 10.1601/strainfinder?urlappend=%3Fid%3DWSM+1271 was included in the analysis. Phylogenetic analysis was done using MEGA, version 6.0 [61] after manually assembling the alignment by using GeneDoc version 2.6.001 [62]. 10.1601/nm.1418 bv biserrulae 10.1601/strainfinder?urlappend=%3Fid%3DWSM+1271 was used as an outgroup and the tree was assembled using the UPGMA algorithm based on the number of nucleotide differences. This approach was used since the potential for genetic recombination among the different 16S rRNA genes as reported by van Berkum [63] cannot be ignored. Bootstrap analysis [64] with 2000 permutations of the data set was done to assess support for the branch points. Strains with a genome sequencing project registered in GOLD [24] are 10.1601/nm.1329 Casida A^T^, 10.1601/nm.1418 bv. biserrulae 10.1601/strainfinder?urlappend=%3Fid%3DWSM+1271 and Mlalz-1 and the GOLD ID is provided in place of the GenBank accession number

Previous studies using multilocus sequence typing showed that *M. laciniata* rhizobia did not form a distinct chromosomal group [15]. Phylogenetic analysis of strain Mlalz-1 was performed by aligning the 16S rRNA sequence (1389 bp from scaffold 84.85) to the 16S rRNA gene sequences of 10.1601/nm.1328 type strains (Fig. 2). Based on four variable sites within this 16S rRNA gene sequence alignment, strain Mlalz-1 is closely related to 10.1601/nm.1335 10.1601/strainfinder?urlappend=%3Fid%3DIAM+12611 ^T^ (= 10.1601/strainfinder?urlappend=%3Fid%3DLMG+6133 ^T^) [21], 10.1601/nm.1334 A 321^T^ (= 10.1601/strainfinder?urlappend=%3Fid%3DLMG+19920 ^T^) [6] and 10.1601/nm.17831 10.1601/strainfinder?urlappend=%3Fid%3DORS+1407 ^T^ [22]. The available IMG 16S rRNA sequence of strain Mlalz-1 gave alignment identities of 100% to 10.1601/nm.1335 10.1601/strainfinder?urlappend=%3Fid%3DIAM+12611 ^T^, 99.7% to 10.1601/nm.1334 A 321^T^ and 99.5% to 10.1601/nm.17831 10.1601/strainfinder?urlappend=%3Fid%3DORS+1407 ^T^. In contrast, 10.1601/nm.1335 10.1601/strainfinder?urlappend=%3Fid%3DIAM+12611 ^T^ and 10.1601/nm.1337 10.1601/strainfinder?urlappend=%3Fid%3DLMG+7834 ^T^ [23] were only 97.3% similar.

## Genome sequencing information

### Genome project history

10.1601/nm.1335 Mlalz-1 was selected for sequencing at the U.S. Department of Energy funded Joint Genome Institute as part of the GEBA-RNB project [17, 18]. The root nodule bacteria in this project were selected based on environmental and agricultural relevance to issues in global carbon cycling, alternative energy production, and biogeochemical importance. In particular, strain Mlalz-1 was chosen since it has strict host specificity for *M. laciniata*, which is suited for cultivation in arid environments [11]. The 10.1601/nm.1335 Mlalz-1 genome project is deposited in the Genomes Online Database [24] and a high-quality permanent draft genome sequence (IMG Genome ID 2513237143) is deposited in IMG [25]. Sequencing, finishing and annotation were performed by the JGI. A summary of the project information is shown in Table 2.

**Table 2:** Genome sequencing project information for 10.1601/nm.1335 Mlalz-1

MIGS ID	Property	Term
MIGS-31	Finishing quality	High-quality permanent draft
MIGS-28	Libraries used	Illumina Standard PE
MIGS-29	Sequencing platforms	Illumina HiSeq 2000
MIGS-31.2	Fold coverage	748×
MIGS-30	Assemblers	Velvet version 1.1.04; ALLPATHS v. r39750
MIGS-32	Gene calling methods	Prodigal 1.4
	Locus Tag	A3CA [78]
	GenBank ID	ATZD00000000
	Genbank Date of Release	January 30 2012
	GOLD ID	Gp0010229 [79]
	BIOPROJECT	165,343
MIGS-13	Source Material Identifier	Mlalz-1 (=10.1601/strainfinder?urlappend=%3Fid%3DUSDA+1984)
	Project relevance	Symbiotic N_2_ fixation, agriculture

### Growth conditions and genomic DNA preparation

10.1601/nm.1335 Mlalz-1 (= 10.1601/strainfinder?urlappend=%3Fid%3DUSDA+1984) was cultured on MAG solid media [26] for three days at 28 °C to obtain well grown, well separated colonies, then a single colony was selected from the plate and inoculated into 5 ml MAG broth media. The culture was grown for 48 h on a gyratory shaker (200 rpm) at 28 °C. Subsequently 1 ml was used to inoculate 50 ml of MAG and the cells were incubated on a gyratory shaker (200 rpm) at 28 °C until an OD_600nm_ of 0.6 was reached. DNA was isolated from 50 ml of cells by Peter van Berkum according to the method described by van Berkum [26]. The final concentration of the DNA was set to 0.5 mg ml^−1^.

### Genome sequencing and assembly

The draft genome of 10.1601/nm.1335 Mlalz-1 was generated at the DOE Joint genome Institute (JGI) using Illumina technology [27]. An Illumina standard PE library was constructed and sequenced using the Illumina HiSeq 2000 platform that generated 35,720,836 reads totalling 4983 Mbp. All general aspects of library construction and sequencing were done at the JGI and details can be found on the JGI website [28]. All raw Illumina sequence data was passed through DUK, a filtering program developed at JGI, which removes known Illumina sequencing and library preparation artefacts (Mingkun L, Copeland A, Han J; unpublished). The following steps for assembly were: (1) filtered Illumina reads were assembled using Velvet (version 1.1.04) [29]; (2) 1–3 Kbp simulated paired end reads were created from Velvet contigs using wgsim (version 0.3.0) [30]; (3) Illumina reads were assembled with simulated read pairs using Allpaths–LG (version r39750) [31]. Parameters for the assembly steps were 1) Velvet: --v --s 51 --e 71 --i 2 --t 1 --f “-shortPaired -fastq $FASTQ” --o “-ins_length 250 -min_contig_lgth 500” for Velvet and 2) wgsim: -e 0–1 76–2 76 -r 0 -R 0 -X 0. The final draft assembly contained 100 contigs in 99 scaffolds. The total size of the genome is 6.7 Mbp and the final assembly is based on 4983 Mbp of Illumina data, which provides an average of 748× coverage of the genome.

### Genome annotation

Genes were identified using Prodigal [32], as part of the DOE-JGI genome annotation pipeline [33, 34]. The predicted CDSs were translated and used to search the National Center for Biotechnology Information non-redundant database, UniProt, TIGRFam, Pfam, KEGG, COG, and InterPro databases. The tRNAScanSE tool [35] was used to find tRNA genes, whereas ribosomal RNA genes were found by searches against models of the ribosomal RNA genes built from SILVA [36]. Other non–coding RNAs such as the RNA components of the protein secretion complex and the RNase P were identified by searching the genome for the corresponding Rfam profiles using INFERNAL [37]. Additional gene prediction analysis and manual functional annotation was done within the Integrated Microbial Genomes-Expert Review platform [38] developed by the Joint Genome Institute, Walnut Creek, CA, USA.

## Genome properties

The genome is 6,664,116 bp with 62.16% GC content (Table 3) and comprised of 99 scaffolds. From a total of 6388 genes, 6314 were protein encoding and 74 RNA only encoding genes. Most genes (79.52%) were assigned a putative function whilst the remaining genes were annotated as hypothetical. The distribution of genes into COGs functional categories is presented in Table 4.

**Table 3:** Genome statistics for 10.1601/nm.1335 Mlalz-1

Attribute	Value	% of Total
Genome size (bp)	6,664,116	100.00
DNA coding (bp)	5,754,332	86.35
DNA G + C (bp)	4,142,407	62.16
DNA scaffolds	99	100.00
Total genes	6388	100.00
Protein-coding genes	6314	98.84
RNA genes	74	1.16
Pseudo genes	0	0.00
Genes in internal clusters	1054	16.50
Genes with function prediction	5080	79.52
Genes assigned to COGs	4659	72.93
Genes with Pfam domains	5317	83.23
Genes with signal peptides	555	8.69
Genes with transmembrane helices	1440	22.54
CRISPR repeats	0	0.00

**Table 4:** Number of genes of 10.1601/nm.1335 Mlalz-1 associated with the general COG functional categories

Code	Value	%age	Description
J	217	4.09	Translation, ribosomal structure and biogenesis
A	0	0.00	RNA processing and modification
K	466	8.77	Transcription
L	122	2.3	Replication, recombination and repair
B	1	0.02	Chromatin structure and dynamics
D	39	0.73	Cell cycle control, cell division, chromosome partitioning
Y	0	0.00	Nuclear structure
V	117	2.20	Defense mechanisms
T	216	4.07	Signal transduction mechanisms
M	301	5.67	Cell wall/membrane/envelope biogenesis
N	72	1.36	Cell motility
Z	0	0.00	Cytoskeleton
W	33	0.62	Extracellular structures
U	74	1.39	Intracellular trafficking, secretion, and vesicular transport
O	206	3.88	Posttranslational modification, protein turnover, chaperones
C	358	6.74	Energy production and conversion
G	555	10.45	Carbohydrate transport and metabolism
E	584	10.99	Amino acid transport and metabolism
F	116	2.18	Nucleotide transport and metabolism
H	242	4.56	Coenzyme transport and metabolism
I	220	4.14	Lipid transport and metabolism
P	279	5.25	Inorganic ion transport and metabolism
Q	159	2.99	Secondary metabolite biosynthesis, transport and catabolism
R	551	10.37	General function prediction only
S	348	6.55	Function unknown
X	36	0.68	Mobilome: prophages, transposons
–	1729	27.07	Not in COGS

## Insights from the genome sequence

10.1601/nm.1335 Mlalz-1 is one of seven strains of 10.1601/nm.1335 that have been sequenced from the GEBA-RNB genome sequencing projects [17]. On the basis of 16S rRNA sequence identity, strain Mlalz-1 is closely related to 10.1601/nm.1335 10.1601/strainfinder?urlappend=%3Fid%3DIAM+12611 ^T^ (= 10.1601/strainfinder?urlappend=%3Fid%3DLMG+6133 ^T^), 10.1601/nm.1334 A 321^T^ (= 10.1601/strainfinder?urlappend=%3Fid%3DLMG+19920 ^T^) and 10.1601/nm.17831 10.1601/strainfinder?urlappend=%3Fid%3DORS+1407 ^T^. As the genomes of these type strains have not been sequenced or are not publically available, gANI values [39] had to be compared with other fully sequenced 10.1601/nm.1328 strains (Table 5). 10.1601/nm.1335 Mlalz-1 currently forms a gANI clique with other 10.1601/nm.1335 strains (gANI values ≥98.14%), compared with gANI values of ≤87.9% with the finished genomes of other 10.1601/nm.1328 strains. This supports the classification of strain Mlalz-1 as an 10.1601/nm.1335 strain, in accordance with the defined species affiliation cut-off value of 96.5% gANI [39]. The total genome size of strain Mlalz-1 is 6.6 Mbp, which falls within the expected size range of 6.6–8.9 Mbp for 10.1601/nm.1335. The genome architecture of 10.1601/nm.1335 consists of a chromosome and the two symbiotic megaplasmids pSymA and pSymB [20]. Replication of a plasmid is initiated by the replication protein encoded by *repC*, which is present as a single copy on 10.1601/nm.1335 pSymA and pSymB. The 10.1601/nm.1335 Mlalz-1 genome carried 2 *repC* loci (A3CADRAFT_00120 and A3CADRAFT_01676) with highest encoded protein identity to RepC proteins of 10.1601/nm.1335 strains. Mlalz-1 A3CADRAFT_00120 RepC1 had highest identity (98.10%) to the RepC1 protein encoded by SMb20044 on pSymB of 10.1601/nm.1335 1021. 10.1601/nm.1335 Mlalz-1 A3CADRAFT_01676 RepC2 had highest identity (99.00%) to the RepC2 protein encoded by SMa2391 on pSymA of 10.1601/nm.1335 1021. This indicated the presence of two megaplasmids in strain Mlalz-1, and revealed that strain Mlalz-1 has a similar genome architecture to that of 10.1601/nm.1335 1021.

**Table 5:** Pairwise gANI comparisons of selected finished genomes of sequenced 10.1601/nm.1328 strains

Strain	Gold ID: Gp	Casida A	USDA 257	WSM 419	1021	AK83	BL225C	GR4	Mlalz-1	Rm41	SM11
*E.adhaerens* Casida A	0094824	100	80.5	79.06	80.12	80.11	80.06	80.01	80.08	80.03	80.06
10.1601/nm.1331 USDA 257	0005169	80.5	100	81.89	83.26	83.24	83.25	83.20	83.14	83.33	83.22
10.1601/nm.1334 WSM419	0000117	79.06	81.93	100	88.18	88.13	88.26	88.24	87.90	88.14	88.26
10.1601/nm.1335 1021	0000726	80.12	83.26	88.19	100	99.36	99.62	99.41	**98.80**	99.24	99.43
10.1601/nm.1335 AK83	0006695	80.08	83.25	88.16	99.36	100	99.33	99.14	**98.60**	99.38	99.33
10.1601/nm.1335 BL225C	0006560	80.06	83.25	88.28	99.62	99.33	100	99.44	**98.81**	99.26	99.39
10.1601/nm.1335 GR4	0020501	80.01	83.23	88.26	99.41	99.14	99.43	100	**98.81**	99.05	99.25
10.1601/nm.1335 Mlalz-1	**0010229**	80.11	83.15	87.91	**98.80**	**98.59**	**99.81**	**98.81**	**100**	**98.59**	**98.66**
10.1601/nm.1335 Rm41	0025853	80.05	83.36	88.11	99.26	99.39	99.25	99.06	**98.59**	100	99.33
10.1601/nm.1335 SM11	0006018	80.05	83.23	88.29	99.45	99.33	99.39	99.26	**98.67**	99.32	100

For 10.1601/nm.1335 Mlalz-1, gANI values above the microbial species delineation cutoff value of 96.5% [39] are in bold font

### Extended insights

**Fig. 3:**
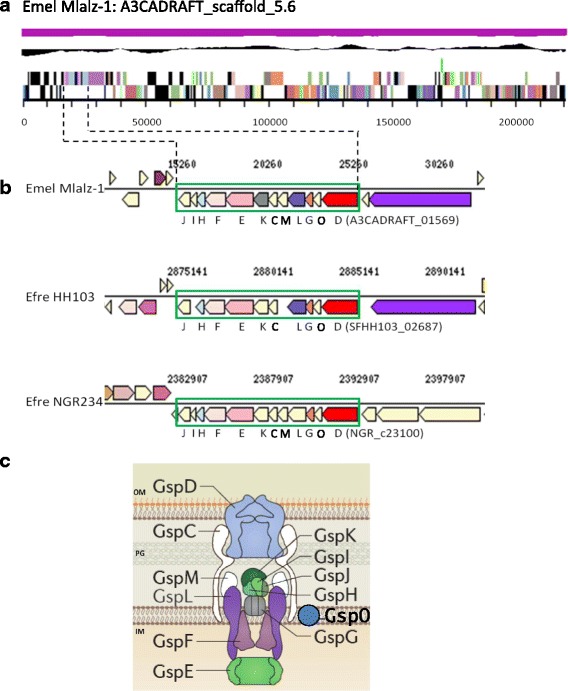
**a** Map of 10.1601/nm.1335 Mlalz-1: A3CADRAFT_scaffold_ 5.6. **b** Genetic organization of the T2SS clusters in 10.1601/nm.1335 Mlalz-1 (*Emel* Mlalz-1), 10.1601/nm.1331 HH103 (*Efre* HH103) and 10.1601/nm.1331 10.1601/strainfinder?urlappend=%3Fid%3DNGR+234 (*Efre* 10.1601/strainfinder?urlappend=%3Fid%3DNGR+234). J, *gspJ*; I, *gspI*; H, *gspH*; F, *gspF*; E, *gspE*; K, *gspK*; C, *gspC*; M, *gspM*; L, *gspL*; G, *gspG*; O, *gspO*; D, *gspD*. **c** Schematics of the T2SS of Gram-negative bacteria [43]. The secretin, GspD (A3CADRAFT_01569); the polytopic protein, GspF (A3CADRAFT_01561); the cytoplasmic ATPase, GspE (A3CADRAFT_01562); the major pseudopilin component, GspG (A3CADRAFT_01567); the minor pseudopilins, GspH (A3CADRAFT_01560), GspI (A3CADRAFT_01559), GspJ (A3CADRAFT_01558) and GspK (A3CADRAFT_01563); the bitopic proteins, GspL (A3CADRAFT_01566), GspC (A3CADRAFT_01564) and GspM (A3CADRAFT_01565); the peptidase, GspO (A3CADRAFT_01568)(GspS is absent from Mlalz-1); OM, outer membrane; PG, peptidoglycan; IM, inner membrane

All 29 10.1601/nm.1335 strains within the gANI clique share a core set of 4948 orthologous genes, using cut off values of 1e-5 and 30% minimum protein identity. 10.1601/nm.1335 Mlalz-1 contains 176 unique genes, 96 (54.5%) of which encode hypothetical proteins. The unique genes include those encoding the components of a T2SS, located on scaffold A3CADRAFT_scaffold_5.6 (Fig. 3a), as well as genes that encode a DNA methyltransferase and a NitT/TauT family transport system. These T2SS components form part of a unique COG profile generated for Mlaz-1 (Table 6). The T2SS secretion system is used to translocate a wide range of proteins from the periplasm across the outer membrane [40]. Although T2SS genes are not found in other 10.1601/nm.1335 strains or in the 10.1601/nm.1331 strains GR64 and 10.1601/strainfinder?urlappend=%3Fid%3DUSDA+257, they are present in the genomes of the 10.1601/nm.1331 strains HH103 and 10.1601/strainfinder?urlappend=%3Fid%3DNGR+234, in a similar gene arrangement to that observed in 10.1601/nm.1335 Mlalz-1 [41, 42] (Fig. 3b). Generally, the T2SS gene cluster is comprised of 12–15 genes, and strain Mlalz-1 contains the 12 required genes *gspDOGLMCKEFHIJ* necessary for a functional T2SS, but lacks the *gspS* gene found only in certain genera [43] (Fig. 3c)*.*

**Table 6:** List of the unique COGs in 10.1601/nm.1335 Mlalz-1

COG	Name	Locus Tag	Gene symbol	Protein function
0393	Uncharacterized conserved protein YbjQ, 10.1601/strainfinder?urlappend=%3Fid%3DUPF+0145 family	A3CADRAFT_01446		Unknown
4970	Tfp pilus assembly protein FimT	A3CADRAFT_01560	*gspH*	T2SS
1459	Type II secretory pathway, component PulF	A3CADRAFT_01561	*gspF*	T2SS
2804	Type II secretory pathway ATPase GspE/PulE or T4P pilus assembly pathway ATPase PilB	A3CADRAFT_01562	*gspE*	T2SS
3156	Type II secretory pathway, component PulK	A3CADRAFT_01563	*gspK*	T2SS
3166	Tfp pilus assembly protein PilN	A3CADRAFT_01566	*gspL*	T2SS
2165	Type II secretory pathway, pseudopilin PulG	A3CADRAFT_01567	*gspG*	T2SS
1450	Type II secretory pathway component GspD/PulD (secretin)	A3CADRAFT_01569	*gspD*	T2SS
2189	Adenine specific DNA methylase Mod	A3CADRAFT_02454	*yhdJ*	DNA methyltransferase
4705	Uncharacterized membrane-anchored protein	A3CADRAFT_05679		Membrane protein
4089	Uncharacterized membrane protein	A3CADRAFT_05685		Membrane protein
2021	Homoserine acetyltransferase	A3CADRAFT_06155		Homoserine acetyltransferase

**Fig. 4:**
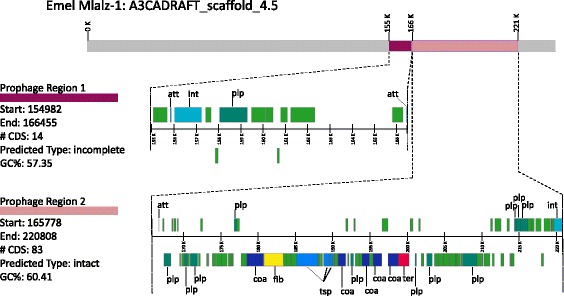
Resident prophages present in 10.1601/nm.1335 Mlalz-1 A3CADRAFT_scaffold_4.5, imaged using PHASTER [44]. Reference locus tag for Prophage Region 1 is A3CADRAFT_01439 (phage capsid family); reference locus tag for Prophage Region 2 is A3CADRAFT_01472 (prophage tail length tape measure protein). Prophage maps not drawn to scale. Attachment site (att), coat protein (coa), fiber protein (fib), integrase (int), phage-like protein (plp), tail shaft protein (tsp), and terminase (ter). All other genes encode hypothetical proteins

In common with some other 10.1601/nm.1335 strains, strain Mlalz-1 contains several genes encoding phage components. The PHASTER algorithm [44] was used to identify two resident prophages, present on scaffold A3CADRAFT_scaffold_4.5: one that was incomplete (Prophage Region 1) and one that was intact (Prophage Region 2) (Fig. 4). The proteins encoded by Prophage Region 1 (11.4 kb) and Prophage Region 2 (55 kb) were most closely related to the phage proteins of PHAGE_Mycoba_Catalina_NC031238 and PHAGE_Sinorh_phiLM21_ NC_029046, respectively.

The Mlaz-1 genome also contains acid-tolerance or acid-responsive genes that are orthologous to the genes identified in the comparatively acid tolerant strain 10.1601/nm.1334 10.1601/strainfinder?urlappend=%3Fid%3DWSM+419. Acid-tolerance or acid-responsive genes identified in Mlaz-1 include *actA (lnt), actP, actR, actS, phrR, exoR, exoH, lpiA, acvB, degP1, mdh3, fbaB, groS, kdpB, kdpC, fixN2* and *fixO2* [45–52] (Additional file 2: Table S2). It is notable that strain Mlalz-1 is unique among the sequenced 10.1601/nm.1328 strains since it contains two versions of the highly acid-induced *lpiA-acvB* operon. One operon (A3CADRAFT_01189-A3CADRAFT_01190) is found on scaffold A3CADRAFT_scaffold_3.4, in a gene region that is conserved in other 10.1601/nm.1335 (sequence similarity >98%) and is located on the chromosome of the fully sequenced 10.1601/nm.1335 1021. The second version of the *lpiA-acvB* operon (A3CADRAFT_05694-A3CADRAFT_05695) is located on A3CADRAFT_scaffold_47.48, in a gene region that is conserved in 10.1601/nm.1334 genomes (sequence similarity >96%) and is located on the pSMED02 symbiotic plasmid of the fully sequenced 10.1601/nm.1334 10.1601/strainfinder?urlappend=%3Fid%3DWSM+419. The regulatory gene *fsrR*, required for the acid activated expression of *lpiA* in 10.1601/nm.1334 10.1601/strainfinder?urlappend=%3Fid%3DWSM+419 [53], is located upstream of A3CADRAFT_05694 in strain Mlalz-1. This regulatory gene is absent from the A3CADRAFT_01190 gene region, and from the *lpiA-acvB* gene regions of all other 10.1601/nm.1335 sequenced genomes. These findings suggest that 10.1601/nm.1335 Mlalz-1 acquired the plasmid-borne *lpiA-acvB* operon and associated *fsrR* regulatory gene by lateral transfer from an 10.1601/nm.1334 strain.

**Fig. 5:**
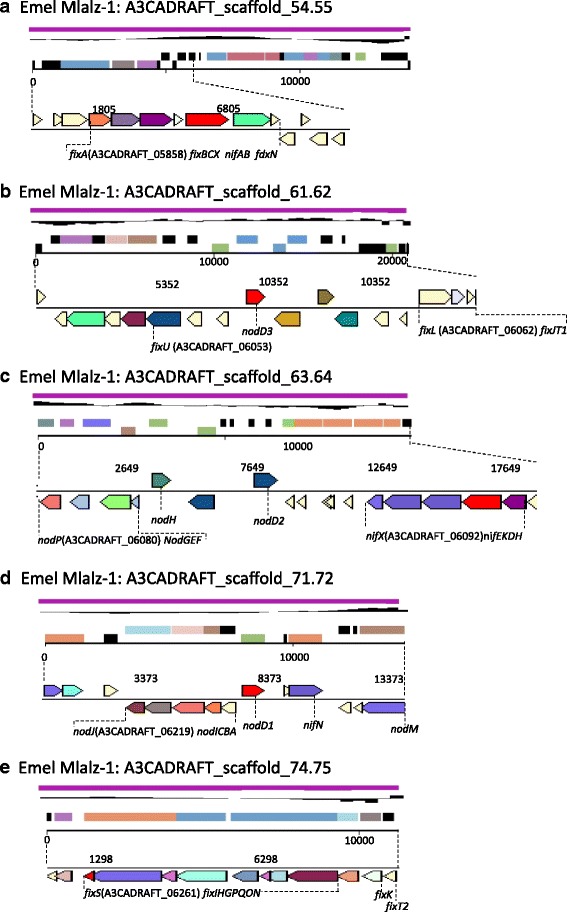
Graphical map of the scaffolds; **a** A3CADRAFT_scaffold_54.55, **b** A3CADRAFT_scaffold_61.62, (**c**) A3CADRAFT_scaffold_63.64, **d** A3CADRAFT_scaffold_71.72 and **e** A3CADRAFT_scaffold_74.75 of 10.1601/nm.1335 Mlalz-1 showing the location of common nodulation (*nod*) and fixation (*nif* and *fix*) genes within the symbiotic regions of this strain. From bottom to the top of the scaffold map: Genes on reverse strand (color by COG categories as denoted by the IMG platform), genes on forward strand (color by COG categories), RNA genes (tRNAs green, sRNAs red, other RNAs black), GC content, GC skew

Essential symbiotic (*nod*, *nif* and *fix*) genes identified in the 10.1601/nm.1335 Mlalz-1 genome (Additional file 2: Table S3 and S4) are located in several clusters on the following scaffolds: A3CADRAFT_scaffold_54.55 (Fig. 5a), A3CADRAFT_scaffold_61.62 (Fig. 5b), A3CADRAFT_scaffold_63.64 (Fig. 5c), A3CADRAFT_scaffold_71.72 (Fig. 5d) and A3CADRAFT_scaffold_74.75 (Fig. 5e). Nodulation of *M. laciniata* has been shown to require a specific *nodC* allele [14]. The *nodC* gene of strain Mlalz-1 has highest sequence identity (≥ 98%) with *nodC* of other *M. laciniata*-nodulating 10.1601/nm.1328 strains in the NCBI database, whereas there is a lower sequence identity (≤ 93%) with *nodC* of 10.1601/nm.1328 strains that nodulate other *Medicago* species. Nodulation of *Medicago* hosts requires Nod factors that are sulfated at the reducing terminus and acylated at the non-reducing terminus, with a polyunsaturated fatty acyl tail [54, 55]. The NodH sulfotransferase, together with the NodP and NodQ sulfate-activating complex, are required for Nod factor sulfation [56, 57]. Activity of NodL results in O-acetylation of the Nod factor [58], while NodE and NodF produce the specific polyunsaturated fatty acyl tail [55, 59]. Strain Mlalz-1 would appear to be typical of 10.1601/nm.1328 strains that nodulate *Medicago* species since the *nodEF*, *nodL* and *nodHPQ* genes that are required for these specific decorations of the Nod factor are present in the genome. 10.1601/nm.1335 Mlalz-1 also possesses the three *nodD* genes that mediate host-specific activation of *nodABC* in the symbiotic interactions of 10.1601/nm.1335 with *Medicago* [60].

## Conclusions

10.1601/nm.1335 Mlalz-1 is a rhizobial strain that is able to nodulate and fix nitrogen with the highly specific host *M. laciniata*. Although the 16S rRNA gene sequence divergence was insufficient to differentiate strain Mlalz-1 from 10.1601/nm.1335 *,* 10.1601/nm.1334 or 10.1601/nm.17831 *,* a gANI value of 98.8% with the genome of 10.1601/nm.1335 1021, compared with 87.9% with the genome of 10.1601/nm.1334 10.1601/strainfinder?urlappend=%3Fid%3DWSM+419 identifies strain Mlalz-1 as 10.1601/nm.1335. Nodulation of *M. laciniata* has been shown to be dependent on the presence of a specific *nodC* allele, which also is present in the genome of 10.1601/nm.1335 Mlalz-1, based on a 98% sequence identity with the *nodC* of other *M. laciniata*-nodulating 10.1601/nm.1328 strains [14]. However, strain Mlalz-1 is unique among sequenced 10.1601/nm.1335 strains in possessing genes encoding components of a T2SS and in having two versions of the adaptive acid tolerance response *lpiA-acvB* operon. The second copy of the 10.1601/nm.1335 Mlalz-1 *lpiA-acvB* operon has highest sequence identity (>96%) with that of sequenced 10.1601/nm.1334 strains, which infers horizontal gene transfer of this region from 10.1601/nm.1334 *.*

## Additional files

Additional file 1: Table S1. Associated MIGS record for *Ensifer meliloti *Mlalz-1. (DOCX 52 kb)

Additional file 2: Table S2-S4. **Table S2**. Acid responsive gene orthologs present in *Ensifer *strains. **Table S3**. The nodulation genes of *Ensifer meliloti *Mlalz-1. **Table S4**. The nitrogen fixation genes of *Ensifer meliloti *Mlalz-1. (DOCX 65 kb)

## Abbreviations

½LA: Half strength Lupin Agar
gANI: Genome-wide average nucleotide identity
GEBA-RNB: Genomic Encyclopedia for Bacteria and Archaea-Root Nodule Bacteria
IMG: Integrated Microbial Genomes
T2SS: Type II Secretion System
TY: Tryptone-yeast extract
