# Supplementary material for: High-quality draft genome sequence of Ensifer meliloti Mlalz-1, a microsymbiont of Medicago laciniata (L.) miller collected in Lanzarote, Canary Islands, Spain
Source: Stand Genomic Sci. 2017 Sep 25;12:58. doi: 10.1186/s40793-017-0270-2 (PMC5613336; doi:10.1186/s40793-017-0270-2)
Supplement: Supplementary file 2 — Table S2. Acid responsive gene orthologs present in Ensifer strains. Table S3. The nodulation genes of Ensifer meliloti Mlalz-1. Table S4. The nitrogen fixation genes of Ensifer meliloti Mlalz-1. (DOCX 65 kb) [file 40793_2017_270_MOESM2_ESM.docx]

Additional file 2

**Table S2** Acid responsive gene orthologs present in *Ensifer* strains.

| **Strain** | **A** | **B** | **C** | **D** | **E** | **F** | **G** | **H** | **I** | **J** | **K** | **L^1^** | **M^2^** | **N** | **O** | **P** | **Q** | **R** | **S** |  |
| --- | --- | --- | --- | --- | --- | --- | --- | --- | --- | --- | --- | --- | --- | --- | --- | --- | --- | --- | --- | --- |
| ***Ensifer arboris*** |  |  |  |  |  |  |  |  |  |  |  |  |  |  |  |  |  |  |  |  |
| LMG 14919^T^ | 0 | 1 | 1 | 1 | 1 | 1 | 1 | 1 | 1 | 1 | 1 | 0 | 1 | 1 | 1 | 1 | 1 | 1 | 1 |  |
|  |  |  |  |  |  |  |  |  |  |  |  |  |  |  |  |  |  |  |  |  |
| ***Ensifer fredii*** |  |  |  |  |  |  |  |  |  |  |  |  |  |  |  |  |  |  |  |  |
| GR64 | 0 | 1 | 1 | 1 | 1 | 1 | 1 | 1 | 1 | 1 | 1 | 0 | 0 | 1 | 1 | 1 | 1 | 1 | 1 |  |
| HH103 | 0 | 1 | 1 | 1 | 1 | 1 | 1 | 1 | 1 | 1 | 1 | 0 | 0 | 1 | 1 | 1 | 1 | 1 | 1 |  |
| NGR234 | 0 | 1 | 1 | 1 | 1 | 1 | 1 | 1 | 1 | 1 | 1 | 0 | 0 | 1 | 1 | 1 | 1 | 1 | 1 |  |
| USDA 257 | 0 | 1 | 1 | 1 | 1 | 1 | 1 | 1 | 1 | 1 | 1 | 0 | 1 | 1 | 1 | 1 | 1 | 1 | 1 |  |
|  |  |  |  |  |  |  |  |  |  |  |  |  |  |  |  |  |  |  |  |  |
| ***Ensifer medicae*** |  |  |  |  |  |  |  |  |  |  |  |  |  |  |  |  |  |  |  |  |
| Di28 | 1 | 1 | 1 | 1 | 1 | 1 | 1 | 1 | 1 | 0 | 0 | 1 | 1 | 1 | 1 | 1 | 1 | 1 | 1 |  |
| WSM1115 | 1 | 1 | 1 | 1 | 1 | 1 | 2 | 1 | 1 | 1 | 1 | 1 | 1 | 1 | 1 | 1 | 1 | 1 | 1 |  |
| WSM1369 | 1 | 1 | 1 | 1 | 1 | 1 | 1 | 1 | 1 | 1 | 1 | 1 | 1 | 1 | 1 | 1 | 1 | 1 | 1 |  |
| WSM244 | 1 | 1 | 1 | 1 | 1 | 1 | 1 | 1 | 1 | 1 | 1 | 1 | 1 | 1 | 1 | 1 | 1 | 1 | 1 |  |
| WSM4191 | 1 | 1 | 1 | 1 | 1 | 1 | 3 | 1 | 1 | 1 | 1 | 1 | 1 | 1 | 1 | 1 | 1 | 1 | 1 |  |
|  |  |  |  |  |  |  |  |  |  |  |  |  |  |  |  |  |  |  |  |  |
| ***Ensifer meliloti*** |  |  |  |  |  |  |  |  |  |  |  |  |  |  |  |  |  |  |  |  |
| 1021 | 0 | 1 | 1 | 1 | 1 | 1 | 2 | 1 | 1 | 1 | 1 | 1 | 1 | 1 | 1 | 1 | 1 | 1 | 1 |  |
| 1A42 | 0 | 0 | 1 | 1 | 1 | 1 | 2 | 1 | 1 | 1 | 1 | 1 | 1 | 1 | 1 | 1 | 1 | 1 | 1 |  |
| 2011 | 0 | 1 | 1 | 1 | 1 | 1 | 2 | 1 | 1 | 1 | 1 | 1 | 1 | 1 | 1 | 1 | 1 | 1 | 1 |  |
| 4H41 | 0 | 1 | 1 | 1 | 1 | 1 | 1 | 1 | 1 | 1 | 1 | 1 | 1 | 1 | 1 | 1 | 1 | 1 | 1 |  |
| 5A14 | 0 | 1 | 1 | 1 | 1 | 1 | 2 | 1 | 1 | 1 | 1 | 1 | 1 | 1 | 1 | 1 | 1 | 1 | 1 |  |
| A0641M | 0 | 1 | 1 | 1 | 1 | 1 | 3 | 1 | 1 | 1 | 1 | 1 | 1 | 1 | 1 | 1 | 1 | 1 | 1 |  |
| A0643DD | 0 | 1 | 1 | 1 | 1 | 1 | 1 | 1 | 1 | 1 | 1 | 1 | 1 | 1 | 1 | 1 | 1 | 1 | 1 |  |
| AE608H | 0 | 1 | 1 | 1 | 1 | 1 | 3 | 1 | 1 | 1 | 1 | 1 | 1 | 1 | 1 | 1 | 1 | 1 | 1 |  |
| AK11 | 0 | 1 | 1 | 1 | 1 | 1 | 1 | 1 | 1 | 1 | 1 | 1 | 1 | 1 | 1 | 1 | 1 | 1 | 1 |  |
| AK58 | 0 | 1 | 1 | 1 | 1 | 1 | 1 | 1 | 1 | 1 | 1 | 1 | 1 | 1 | 1 | 1 | 1 | 1 | 1 |  |
| AK75 | 0 | 1 | 1 | 1 | 1 | 1 | 1 | 1 | 1 | 1 | 1 | 1 | 1 | 1 | 1 | 1 | 1 | 1 | 1 |  |
| AK83 | 0 | 1 | 1 | 1 | 1 | 1 | 4 | 1 | 1 | 1 | 1 | 1 | 1 | 1 | 1 | 1 | 1 | 1 | 1 |  |
| BL225C | 0 | 1 | 1 | 1 | 1 | 1 | 2 | 1 | 1 | 1 | 1 | 1 | 1 | 1 | 1 | 1 | 1 | 1 | 1 |  |
| BO21CC | 0 | 1 | 1 | 1 | 1 | 1 | 2 | 1 | 1 | 1 | 1 | 1 | 1 | 1 | 1 | 1 | 1 | 1 | 1 |  |
| C0431A | 0 | 1 | 1 | 1 | 1 | 1 | 2 | 1 | 1 | 1 | 1 | 1 | 1 | 1 | 1 | 1 | 1 | 1 | 1 |  |
| C0438LL | 0 | 0 | 1 | 1 | 1 | 1 | 1 | 1 | 1 | 1 | 1 | 1 | 1 | 1 | 1 | 1 | 1 | 1 | 1 |  |
| CCNWSX0020 | 0 | 1 | 1 | 1 | 1 | 1 | 1 | 1 | 1 | 1 | 1 | 1 | 1 | 1 | 1 | 1 | 1 | 1 | 1 |  |
| CIAM1775 | 0 | 1 | 1 | 1 | 1 | 1 | 1 | 1 | 1 | 1 | 1 | 1 | 1 | 1 | 1 | 1 | 1 | 1 | 1 |  |
| GR4 | 0 | 1 | 1 | 1 | 1 | 1 | 3 | 1 | 1 | 1 | 1 | 1 | 1 | 1 | 1 | 1 | 1 | 1 | 1 |  |
| GVPV12 | 0 | 1 | 1 | 1 | 1 | 1 | 1 | 1 | 1 | 1 | 1 | 0 | 1 | 1 | 1 | 1 | 1 | 1 | 1 |  |
| H1 | 0 | 1 | 1 | 1 | 1 | 1 | 1 | 1 | 1 | 1 | 1 | 1 | 1 | 1 | 1 | 1 | 1 | 1 | 1 |  |
| Mlalz-1 | 0 | 1 | 1 | 1 | 1 | 1 | 3 | 1 | 1 | 2 | 2 | 1 | 1 | 1 | 1 | 1 | 1 | 1 | 1 |  |
| MVII-I | 0 | 1 | 1 | 1 | 1 | 1 | 1 | 1 | 1 | 1 | 1 | 1 | 1 | 1 | 1 | 1 | 1 | 1 | 1 |  |
| Rm41 | 0 | 1 | 1 | 1 | 1 | 1 | 1 | 1 | 1 | 1 | 1 | 1 | 1 | 1 | 1 | 1 | 1 | 1 | 1 |  |
| RRI128 | 0 | 1 | 1 | 1 | 1 | 1 | 1 | 1 | 1 | 1 | 1 | 1 | 1 | 1 | 1 | 1 | 1 | 1 | 1 |  |
| SM11 | 0 | 1 | 1 | 1 | 1 | 1 | 2 | 1 | 1 | 1 | 1 | 1 | 1 | 1 | 1 | 1 | 1 | 1 | 1 |  |
| WSM1022 | 0 | 1 | 1 | 1 | 1 | 1 | 1 | 1 | 1 | 1 | 1 | 1 | 1 | 1 | 1 | 1 | 1 | 1 | 1 |  |
|  |  |  |  |  |  |  |  |  |  |  |  |  |  |  |  |  |  |  |  |  |
| ***Ensifer sp.*** |  |  |  |  |  |  |  |  |  |  |  |  |  |  |  |  |  |  |  |  |
| BR816 | 0 | 1 | 1 | 1 | 1 | 1 | 1 | 1 | 1 | 1 | 1 | 0 | 0 | 1 | 1 | 1 | 1 | 1 | 1 |  |
| PC2 | 0 | 1 | 1 | 1 | 1 | 1 | 1 | 1 | 1 | 1 | 1 | 0 | 1 | 1 | 1 | 1 | 1 | 1 | 1 |  |
| TW10 | 0 | 1 | 1 | 1 | 1 | 1 | 1 | 1 | 1 | 1 | 1 | 0 | 1 | 1 | 1 | 1 | 1 | 1 | 1 |  |
| USDA6670 | 0 | 1 | 1 | 1 | 1 | 1 | 1 | 1 | 1 | 1 | 1 | 0 | 1 | 1 | 1 | 1 | 1 | 0 | 1 |  |
| WSM1721 | 0 | 1 | 1 | 1 | 1 | 1 | 1 | 1 | 1 | 1 | 1 | 0 | 1 | 1 | 1 | 1 | 1 | 1 | 1 |  |
| A=Hypothetical protein (Smed_6030), B=Protein of unknown function DUF892 (Smed_3491), C=Protease Do (Smed_0637), D=Malate dehydrogenase (Smed_2944), E=Fructose-bisphosphate aldolase (Smed_2654), F=Phasin (Smed_0319), G=Chaperonin Cpn10 (Smed_6083/Smed_0409), H=Regulator (Smed_0045), I=17 kDa surface protein (Smed_0339), J=Transmembrane protein (Smed_5951), K=Acid virulence protein (Smed_5950), L=ABC transporter (Smed_3820), M=ABC transporter (Smed_5319), N=Lipopolysaccharide biosynthesis protein (Smed_4573), O=Potassium-importing ATPase (Smed_5359), P=Potassium-importing ATPase (Smed_5360), Q=Cytochrome *cbb_3_* oxidase, subunit I (Smed_5935), R=Cytochrome *cbb_3_* oxidase, subunit II (Smed_5934), S=DNA ligase (Smed_4303),^1^= consist of 4 genes in the operon, ^2^= consist of 5 genes in the operon. | | | | | | | | | | | | | | | | | | | | |

**Table S3** The nodulation genes of *Ensifer meliloti* Mlalz-1.

| **Locus Tag** | **Gene** | **Protein** | **Scaffolds** |
| --- | --- | --- | --- |
| A3CADRAFT_00880 | *nfeD* | membrane-bound serine protease (ClpP class) | A3CADRAFT_scaffold_2.3 |
| A3CADRAFT_01227 | *nodG* | 3-oxoacyl-(acyl-carrier-protein) reductase | A3CADRAFT_scaffold_3.4 |
| A3CADRAFT_02165 | *nolO* | carbamoyltransferase | A3CADRAFT_scaffold_8.9 |
| A3CADRAFT_03411 | *nolR* | nodulation repressor NolR | A3CADRAFT_scaffold_16.17 |
| A3CADRAFT_05078 | *nodM* | glucosamine synthase | A3CADRAFT_scaffold_35.36 |
| A3CADRAFT_05146 | *nodP2* | sulfate adenylyltransferase subunit 2 | A3CADRAFT_scaffold_36.37 |
| A3CADRAFT_05697 | *nodQ2* | adenylylsulfate kinase /sulfate adenylyltransferase subunit 1 | A3CADRAFT_scaffold_48.49 |
| A3CADRAFT_05882 | *noeB* | Nodulation protein NoeB | A3CADRAFT_scaffold_54.55 |
| A3CADRAFT_05883 | *noeB* | Nodulation protein NoeB (portion) |  |
| A3CADRAFT_06056 | *nodD3* | transcriptional regulator; lysR family | A3CADRAFT_scaffold_61.62 |
| A3CADRAFT_06080 | *nodP1* | sulfate adenylyltransferase subunit 2 | A3CADRAFT_scaffold_63.64 |
| A3CADRAFT_06081 | *nodG* | 3-oxoacyl-(acyl-carrier-protein) reductase |  |
| A3CADRAFT_06082 | *nodE* | 3-oxoacyl-(acyl-carrier-protein) synthase |  |
| A3CADRAFT_06083 | *nodF* | acyl carrier protein |  |
| A3CADRAFT_06084 | *nodH* | sulfotransferase |  |
| A3CADRAFT_06086 | *nodD2* | transcriptional regulator; lysR family |  |
| A3CADRAFT_06216 | *nodQ1* | sulfate adenylate transferase | A3CADRAFT_scaffold_71.72 |
| A3CADRAFT_06219 | *nodJ* | membrane transport nodulation protein, ABC-type transporter |  |
| A3CADRAFT_06220 | *nodI* | Nod factor export transport ATP-binding protein I (Nodulation ATP- binding protein I) |  |
| A3CADRAFT_06221 | *nodC* | N-acetylglucosaminyltransferase |  |
| A3CADRAFT_06222 | *nodB* | chitooligosaccharide deacetylase |  |
| A3CADRAFT_06223 | *nodA* | N-acyltransferase |  |
| A3CADRAFT_06224 | *nodD1* | transcriptional regulator; lysR family |  |
| A3CADRAFT_06229 | *nodM* | glucosamine synthase |  |
| A3CADRAFT_06260 | *nodL* | Acetyltransferase | A3CADRAFT_scaffold_74.75 |
| A3CADRAFT_06397 | *noeA* | Ribosomal protein L11 methylase | A3CADRAFT_scaffold_96.97 |

**Table S4** The nitrogen fixation genes of *Ensifer meliloti* Mlalz-1.

| **Locus Tag** | **Gene** | **Protein** | **Scaffold** |
| --- | --- | --- | --- |
| A3CADRAFT_05218 | *nifR* | tRNA-dihydrouridine synthase | A3CADRAFT_scaffold_37.38 |
| A3CADRAFT_05858 | *fixA* | electron transfer flavoprotein beta-subunit | A3CADRAFT_scaffold_54.55 |
| A3CADRAFT_05859 | *fixB* | electron transfer flavoprotein, alpha subunit |  |
| A3CADRAFT_05860 | *fixC* | electron-transferring-flavoprotein dehydrogenase |  |
| A3CADRAFT_05861 | *fixX* | ferredoxin-like protein |  |
| A3CADRAFT_05862 | *nifA* | transcriptional activator |  |
| A3CADRAFT_05863 | *nifB* | FeMo cofactor biosynthesis protein |  |
| A3CADRAFT_05864 | *fdxN* | ferredoxin |  |
| A3CADRAFT_06053 | *degT/ fixU* | nitrogen fixation protein | A3CADRAFT_scaffold_61.62 |
| A3CADRAFT_06062 | *fixL* | two-component system, LuxR family, sensor kinase FixL |  |
| A3CADRAFT_06063 | *fixJ* | two component transcriptional regulator, LuxR family |  |
| A3CADRAFT_06064 | *fixT1* | FixT1 Inhibitor of FixL autophosphorylation |  |
| A3CADRAFT_06091 | *fdxB* | ferredoxin iron-sulfur 4Fe-4S | A3CADRAFT_scaffold_63.64 |
| A3CADRAFT_06092 | *nifX* | nitrogen fixation protein |  |
| A3CADRAFT_06093 | *nifE* | oxidoreductase, nitrogenase iron-molybdenum cofactor biosynthesis protein |  |
| A3CADRAFT_06094 | *nifK* | nitrogenase molybdenum-iron protein beta chain (nitrogenase component I) (dinitrogenase) |  |
| A3CADRAFT_06095 | *nifD* | nitrogenase molybdenum-iron protein alpha chain (nitrogenase component I) (dinitrogenase) |  |
| A3CADRAFT_06096 | *nifH* | nitrogenase Fe protein (nitrogenase component II) (dinitrogenase reductase) |  |
| A3CADRAFT_06226 | *nifN* | nitrogenase molybdenum-iron protein NifN | A3CADRAFT_scaffold_71.72 |
| A3CADRAFT_06261 | *fixS* | cytochrome oxidase maturation protein | A3CADRAFT_scaffold_74.75 |
| A3CADRAFT_06262 | *fixI* | Cu2+-exporting ATPase |  |
| A3CADRAFT_06263 | *fixH* | nitrogen fixation protein |  |
| A3CADRAFT_06264 | *fixG* | cytochrome c oxidase accessory protein |  |
| A3CADRAFT_06265 | *fixP* | cytochrome c oxidase cbb3-type subunit 3 |  |
| A3CADRAFT_06266 | *fixQ* | cytochrome c oxidase cbb3-type subunit 4 |  |
| A3CADRAFT_06267 | *fixO* | cytochrome c oxidase cbb3-type subunit 2 |  |
| A3CADRAFT_06268 | *fixN* | cytochrome c oxidase cbb3-type subunit 1 |  |
| A3CADRAFT_06270 | *fixK* | CRP/FNR family transcriptional regulator, nitrogen fixation regulation protein |  |
| A3CADRAFT_06271 | *fixT2* | FixT2 Inhibitor of FixL autophosphorylation |  |
